# 

**Published:** 1887-01-01

**Authors:** 


### The Hospital.

**An Institution, Family, and Parochial Journal of Hospitals, Asylums, and all Agencies for the Care of the Sick, Criticism and News.**

SATURDAY, JANUARY 1ST, 1887.

### THE IN- AND OUT-PATIENTS' HOSPITAL DIARY.

This Diary is so arranged as to make some portion of it appear every week, and the whole in each monthly part. It has been very difficult to compile, and corrections will at all times be gratefully received. It has been suggested that similar information about the larger Provincial and Scotch Hospitals would be useful to many readers. We should be glad to hear if this is felt to be the case.

### I.—GENERAL HOSPITALS.

| Hospitals and Terms of Admission. | Physicians, Surgeons, and Medical Officers, with Days and Hours of Attendance. |
|---|---|
| CHARING CROSS HOSPITAL, West Strand, W.C. Sec., Mr. A. E. Reade<br>Urgent cases are immediately admitted as in-patients. Subscriber's letter expected in other cases<br>Out-patients admitted without subscriber's letter first time; for continued attendance they must be obtained from the Secretary | *In-Physicians*, Drs. Pollock, M. Th.; Green, W. S.; Bruce, Tu. F. Hour, 1.30<br>*In-Surgeons*, Messrs. Barwell, M. Th.; Bellamy, Tu. F.; Bloxam, W. S. Hour, 2<br>*Out-Physicians*, Drs. Wilcocks, M.Th.; Abercrombie, Tu. F.; Lubbock, W. S.; Murray, W. S. Hour 1.30<br>*Out-Surgeons*, Messrs. Cantlie, M.Th.: J. H. Morgan, Tu. F.; Stanley Boyd, W. S. Hour, 1.30 |
| "DREADNOUGHT" HOSPITAL (Seamen's Hospital Society), Greenwich, S.E. Sec., Mr. W. T. Evans<br>Entirely free to seamen and to all accident cases | *In- and Out-Physicians*, Drs. Curnow, Tu. Th. S.; H. T. Griffiths, M.W.F. Daily, between 9 and 3<br>*In- and Out-Surgeons*, Messrs. W. J. Smith, G. R. Turner. Daily, between 9 and 3 |
| FRENCH HOSPITAL, 10, Leicester Place, Leicester Sq., W.C. Sec., Mr. E. Rimmel<br>Free to urgent cases and to all foreigners speaking French | *In-Physician*, Dr. Vintras, Tu. S. 10<br>*In-Surgeons*, Sir W. MacCormac, Tu. 9; Mr. J. Késer, W. F. 10<br>*Out-Physician*, Dr. Vintras, Tu. S. 10<br>*Out-Surgeons*, Messrs. H. de Méric, M. Th. 10; J. Késer, W. F. 10 |
| GERMAN HOSPITAL, 113, Dalston Lane, E. Sec., Mr. C. Feldmann<br>Free to natives of Germany, and others speaking German, and to all accidents and emergencies. Others by Governor's letters<br>——— Eastern Branch, 49, Mansell Street, Aldgate, E.<br>——— Western Branch, 259, Oxford Street, W. | *In-Physicians*, Drs. Weber, M. T. 2 Port, Tu. F. 9 a.m.<br>*In-Surgeons*, Drs. Lichtenberg and Burger, W. S. 2<br>*Out-Physicians*, Drs. Tuchmann, Tu. W. 2; Ludwig, M. Th. 2; Keller, Tu. W. F. 2; Philippi, M. Th. F. 2 (*Men*, Tu. Th.; *Women*, M. W. F.)<br>*Out-Physician*, Dr. C. Harrer, Tu. F. 8 to 10 a.m.<br>*Out-Physician*, Dr. M. Castaneda, M. T. 8 to 10 a.m. |
| GREAT NORTHERN CENTRAL HOSPITAL, Caledonian Road, N. Sec., Mr. W. T. Grant<br>Free to all, without letters of recommendation | *In-Physicians*, Drs. Cholmeley, Cook, and Burnett, M. Tu. W. Th. F. 2<br>*In-Surgeons*, Messrs. W. Adams, W. Spencer Watson, and J. Macready, M. Tu. W. Th. F. 2<br>*Out-Physicians*, Drs. Beale, M. Th. 2; Beevor, Tu. 2, S. 10<br>*Out-Surgeons*, Messrs. Lockwood, M. Th. 2; Makins, Tu. F. 2 |
| GUY'S HOSPITAL, St. Thomas's Street, S.E. Supt., Dr. C. J. Steele<br>Patients are admitted, if beds are available, without letters of recommendation, on application at the Hospital. Out-patients are seen free without letters of recommendation | *In-Physicians*, Drs. Pavy, Tu. F.; Pye-Smith, M. Th. S.; Taylor, M. Tu. Th. F. Hour, 1.30<br>*In-Surgeons*, Messrs. Bryant, M. Th. Durham, M. Th. F.; Howse, W. S.; D. Colley, M. Th. Hour, 1.30<br>*Out-Physicians*, Drs. Carrington, W.; Hale White, M.; Goodhart, M. Tu. Th. F. Hour, 12.30<br>*Out-Surgeons*, Messrs. Lucas, Th.; Golding Bird, S.; Jacobson, W.; Symonds, M. Hour, 12.30 |
| KING'S COLLEGE HOSPITAL, Portugal Street, W.C. Sec., Mr. T. Mosse Macdonald<br>Accidents and urgent cases free at all times. Other cases by Governors' letters, but where not obtainable, application may be made to the Secretary | *In-Physicians*, Drs. Beale, Duffin, Yeo, Tu. W. F. 1.30; Tu. W. F. S. 2<br>*In-Surgeons*, Mr. Wood, Sir J. Lister, Messrs. H. Smith, Cheyne, W. Rose, Barrow. Daily, 1.30<br>*Out-Physicians*, Drs. Ferrier, J. Curnow, N. I. C. Tirard; *Men*, Tu.Th.; S. 1; *Women*, M. W. F. 1<br>*Out-Surgeons*, Messrs. W. Rose, W. Cheyne; *Men*, Tu. Th. S. 1; *Women*, M. W. F. 1 |
| LONDON HOSPITAL, Whitechapel Road, E. Sec., Mr. A. Haggard<br>Accidents and urgent cases free; out-patients for dental and maternity departments need no recommendation; other cases by subscriber's order. Out-patients are required to attend only once, unless otherwise directed by the Physician or Surgeon | *In-Physicians*, Drs. Mackenzie, Tu. F.; Down, Tu. F.; H. Jackson, M. Th.; Sutton, M. Th.; Fenwick, Tu. F. 1.30<br>*In-Surgeons*, Messrs. Rivington, Tu. F.; Couper, Waren Tay, M. Th.; McCarthy, W.S.; Treves, Tu.F. Hour, 1.30<br>*Out-Physicians*, Drs. Sansom, Th.; M. Ralfe, M. Th.; Turner, Tu. F.; Warner, Tu. F.; Gilbart Smith, W. S. J. Anderson, W. S. Hour, 1.30<br>*Out-Surgeons*, Messrs. Mansell-Moullin, M. Th.; Eve, M. W.; Reeves, Tu. S.; H. Fenwick, Tu.F. Hour, 1.30 |

| Hospitals and Terms of Admission. | Physicians, Surgeons, and Medical Officers, with Days and Hours of Attendance. |
|---|---|
| LONDON TEMPERANCE HOSPITAL, Hampstead Road, N.W.<br>For treatment without alcohol. By letter or small payment | *In- and Out-Physicians*, Drs. J. Edmunds, M.; J. J. Ridge, Tu. F. Hour, 1.30<br>*In- and Out-Surgeons*, Mr. A. P. Gould, Th. 2.30 |
| METROPOLITAN FREE HOSPITAL, Kingsland Road, N. Sec., Mr. G. Croxton<br>Admission free to the sick poor, without any letter of recommendation. Urgent cases at all times | *In- and Out-Physicians*, Drs. Drysdale, Tu. F. 12; J. G. Dudley, W. S. 12; Fotherby, M. Th. 12; H. H. Tooth, Tu. F. 9; A. Haig, W. S. 9; A. Davies, M. Th. 9<br>*In- and Out-Surgeons*, Messrs. E. J. Chance, W. S. 9; Goodsall, Tu. F. 9; Walsham, M. Th. 9; A. A. Bowlby, F. 9; S. Paget, Th. 9; C. J Thompson, daily, 9 |
| MIDDLESEX HOSPITAL, Berners Street, W. Sec. and Supt., Mr. A. O. D. Bartholeyns<br>Urgent cases admitted at all times; other cases by ticket. All medical cases must, in the first instance, be seen by the Resident Medical Officer, who attends at the hospital daily, at 11 a.m. | *In-Physicians*, Drs. Cayley, M. Th.; Coupland, Tu. F.; D. Powell, M.Th.; Finlay, Tu. F. Hour, 1.30<br>*In-Surgeons*, Messrs. Hulke, M. Th.; Lawson, M. Th.; Morris, Tu. F. Hour, 1.30<br>*Out-Physicians*, Drs. Fowler, Tu. F. 3.30; Biss, M. Th. 1.30; Pringle, W. S. 1.30<br>*Out-Surgeons*, Messrs. Andrew Clark, M. 1.15 (*Men*); Th. 1.15 (*Women*): Pearce Gould, F. 1.30 (*Men*); Tu. 1.30 (*Women*); J. B. Sutton, W. S. 9 |
| MILLER HOSPITAL AND KENT DISPENSARY, Greenwich, S.E. | *Physicians*, Drs. T. Creed, C. H. Hartt, G. Willes<br>*Surgeons*, Messrs. T. Moore, J. Poland, E. Clarke |
| NORTH-WEST LONDON HOSPITAL, 18, Kentish Town Road, N.W. Sec., Mr. A. Craske<br>By subscribers' tickets or payment, but accidents and urgent cases free at all times | *In- and Out-Physicians*, Drs. W. C. Hood, J. Shaw, Edwarde H. Campbell. Daily, 1.30<br>*In- and Out-Surgeons*, Messrs. F. Durham, Collier, Jennings, J. Black. Daily, 1.30 |
| OSPEDALE ITALIANO, 41, Queen Square, Bloomsbury. Sec., Sig. L. Bonacina<br>Free to all, but Italians have preference | *In- and Out-Physicians*, Drs. M. Castaneda, Tu. F. 10 to 11; B. Sassella, M.Th. 10 to 11; Mr. J.W.B. Steggall, W.S. 4 to 5 |
| POPLAR HOSPITAL, 303, East India Dock Road, E. Sec., Lt.-Col. Feneran<br>Free to accidents and emergencies | *In-Surgeons*, Messrs. F. M. Corner, M. Brownfield, and Resident Surgeons<br>*Out-Surgeons*, Dr. J. D. Grant, W. S. Mr. T. E. Bowkett, Tu. F. 12 to 2 |
| ROYAL FREE HOSPITAL, Gray's Inn Road, W.C. Sec., Mr. J. S. Blyth<br>Admission free to the sick poor, without any letter of recommendation | *In- and Out-Physicians*, Drs. Cockle, M. Th.; Sainsbury, Tu. F.; West, W. S. Hour, 1<br>*In- and Out-Surgeons*, Messrs. Rose, M. Th.; W. Gant, Tu. F.; Barrow, W. S. Hour, 1 |
| ST. BARTHOLOMEW'S HOSPITAL, Smithfield. Clerk, Mr. W. Cross<br>Admission free, without letter or ticket. Accidents and other urgent cases admitted at any time; ordinary cases of disease daily, between 9 and 10. Patients are seen for Electrical treatment on M. Tu. Th. and F. 1.30 | *In-Physicians*, Drs. Andrew, Church, Gee, Sir D. Duckworth. Daily, 1.30<br>*In-Surgeons*, Messrs. Savory, T. Smith, Willett, Langton, M. Baker. Daily, 1.30<br>*Out-Physicians*, Drs. Hensley, M. Th. 11; Brunton, Tu. F. 11; Legg, W. S. 11; N. Moore, Tu. W. Th. S. 9<br>*Out-Surgeons*, Messrs. Marsh, Tu. F.; 12; Butlin, M. Th. 12; Walsham, W. S. 12; Cripps and Bruce Clarke, daily, 9<br>*Casualty Physicians*, Drs. Davies, Tu. W. F. S.; O. Browne, M. Tu. Th. F.; Nias, M. W. Th. S. Hour, 9 |
| ST. GEORGE'S HOSPITAL, Hyde Park Corner, S.W. Sec., Mr. C. L. Todd<br>Free to accidents and emergencies. Other in-cases by Governor's or subscriber's letter, but, in necessitous cases, this recommendation is not strictly insisted on. Letters are not required for out-patients. Vaccination on Th. at 11 | *In-Physicians*, Drs. Wadham, M. F.; Dickinson, M. Tu. Th. F.; Whipham, Tu.W. S.; Cavafy, Tu. S. Hour, 1<br>*In-Surgeons*, Messrs. Holmes, M. Th, F. 1, W. 1.30: Rouse, M. Th. F. 1, W. 1.30; Haward, Tu. Th. S. 1, W. 1.30<br>*Out-Physicians*, Drs. Ewart, M. F.; Owen, Tu. S. Hours, 10.30 to 12<br>*Out-Surgeons*, Messrs. Bennett, M. F.; Dent, Tu. S. Hours, 10.30 to 12 *Men*, F. S.; *Women*, M. Tu. |

### THE CHILDREN'S CORNER.

TO-DAY we commence the second of our quarterly prize competitions. The Puzzle Editor trusts that all his "old" competitors (successful or unsuccessful in the last) will again enter the lists, while he hopes to have the pleasure of receiving answers from many little people who have not yet taxed their powers of guessing. Might he ask his present little contributors to invite their friends? The names of the Prize Winners in the First Quarterly Competition will appear next week.

### OUR PASTIMES.

**Children's Quarterly Prize Competition.**

| | |
|---|---|
| Begins .. .. .. .. | 1st Jan, 1887 |
| Ends .. .. .. .. | 26th Mar., 1887 |

PRIZES.

| | |
|---|---|
| 1st .. .. .. .. | Thirty Shillings |
| 2nd .. .. .. .. | Twenty " |
| 3rd .. .. .. .. | Ten " |
| 4th .. .. .. .. | Five " |

will be awarded to the four competitors who shall have sent in during the thirteen weeks, the largest number of correct answers to our pastimes.

**First Competition.**

No. 1. BURIED PROVERBS. Each of the following sentences contains a word of a well-known proverb. "A" is an article. Little girls should learn the way to stitch. Snow lies on the ground in winter. There is a time for everything. The little boy saves his money. Three threes make nine.

No 2. BEHEADED WORDS.

My whole is clever, full of dextrous pow'r,
Behead me, I of slaughter tell,
Behead again, and then, unhappy hour,
I'm just the opposite of well.

No. 3. REBUS. Can any of my little friends tell me why a young lady is like a Bill of Exchange?

No. 4. DIAGONAL PUZZLE.

```
* . . . *
  * . *
    *
  * . *
* . . . *
```

The first and last lines give the names of two celebrated French rivers. Read downwards from left, you again get the name of the first river; read upwards from the left, you again get the second river. The lights are: in line two, a bird; in line three, an interjection; in line four, the author of a famous French exercise book.

No. 5. DOUBLE ACROSTIC. The reverse of old; a girl's Christian name; the noise a lion makes; a synonym for fairies. My initials are indispensable in a hospital; and my finals are incomplete without them.

No. 6. REBUS. Who can tell me why a Latin or a Greek grammar resembles a hospital?

No. 7. SQUARE PUZZLE. I want you to arrange the undermentioned symbols in such order that, reading the lines horizontally, vertically, or diagonally, one of each of the four numbers, and of each of the four letters shall be found. The symbols must, of course, be arranged in a square.

| | | | |
|---|---|---|---|
| 1a | 1b | 1c | 1d |
| 2a | 2b | 2c | 2d |
| 3a | 3b | 3c | 3d |
| 4a | 4b | 4c | 4d |

**Results of Twelfth Competition.**

No. 61. The longest day to Adam in Paradise must have been that which saw no *Eve!* All my little friends guessed this.

No. 62. So, too, they all unearthed the "buried towns" by bringing "to light" Rome, Naples, and Tyre.

No. 63. Only Tina here: this was very sad, for I am sure the puzzle was not a hard one. *Je bis à vore*, is, of course, *Je bois sans eau à votre santé*. You get it thus: *bis* is without its "o" (=*bois sans eau*), while *vore* is without its "*t*" (=*votre sans* "*t*," or *votre santé*).

No. 64. Here was a very easy one guessed all round. "Adam, Seth, Eve, Cain, Abel" (Adam, saith Eve, cane Abel).

No. 65. Another easy one! The letter was meant for "His Excellency Sir Humphrey Davy."

No. 66. The *lights* in this square word are

S P R I N G
P R I M E R
R I P P L E
I M P O S E
N E L S O N
G R E E N S

Here you see each line read vertically, gives the same word as the corresponding line read horizontally. Retsibsi errs in giving "intake" for "impose," while Alsie writes "inveigle." These words would not, of course, meet this condition.

No 67. The answer here is "money," "wealth," or "riches." These we all like, though they sometimes bring people to "bad ends." Mikado, evidently in a vein of quiet irony anent the "bad ends" which over-indulgence in the good things of Christmas produces, suggests "A Merry Christmas!"

All right—None.

Six right—T. K., Florrie, Tina, Jimmy.

Five right—Retsibsi, Mikado.

Four right—Alsie, Little Mary, True Blue, Filomel, Bismarck.

Answers, having the actual name and address (to which a pseudonym may be added for publication), must have "The Children's" Coupon attached (see advertisement pages), and be addressed to the 'PUZZLE EDITOR," THE HOSPITAL, Norfolk House, Norfolk Street, Strand, W.C., not later than Thursday, 6th January, 1887. Results will appear in our issue of the 15th January, 1887.

### "THE HOSPITAL" CHARITY BOX.

TO OUR READERS.

WE regret to have to inform our readers that the object for which we started our "Charity Box" Competition has not been attained. In devising this feature of THE HOSPITAL, we anticipated that, while giving our readers an opportunity for a little friendly competition and amusement, we should at the same time have been enabled to afford "a little practical help to the hospitals." We shall publish next week a tabulated return showing the result of the "Charity Box" during the past thirteen weeks, from which it will be seen that on one occasion only did the entrance fees counterbalance the amount we have been giving weekly in prizes. Under these circumstances, it has appeared to us best to close our "Charity Box." In doing so, we can only say that we have done our utmost to make the awards fairly and impartially, and, though one or two correspondents have frowned upon us because they did not win prizes at the second or third attempt, we have received a large number of letters from others (many non-winners) testifying to their complete satisfaction with the manner in which we have conducted this competition.

RESULTS OF TWELFTH COMPETITION.

The "Patient" Competition has resulted as follows:—

| | Accepted Score. | |
|---|---|---|
| Mr. R. Greene, County Asylum, Northampton .. .. .. .. .. .. .. | 91 | 1st and 2nd Prizes, a "Tie." |
| Miss Goldsmith, 60, Portland Place, W. .. | 91 | |
| Miss Laura Bainbridge, Ashton House, St. Helier's, Jersey .. .. .. .. .. | 86—3rd Prize. | |

### NOTICE TO CORRESPONDENTS.

All correspondence must be written on one side of the paper only. We cannot undertake to return MSS. not used.

**Communications,** whether intended for insertion or for private information, must be authenticated by the names and addresses of the writers, not necessarily for publication.

**Local papers** containing reports or news paragraphs **should be marked.**

It is especially requested that early intelligence of **local events of interest,** or which it may be thought desirable to bring under notice, may be sent direct to the Editor.

Communications respecting editorial matters should be addressed to the Editor, Norfolk House, Norfolk Street, Strand, London, W.C.

### BUSINESS COMMUNICATIONS AND ADVERTISEMENTS.

All communications concerning business matters, non-delivery of THE HOSPITAL, etc., should be addressed to the Manager, at the Publishing Offices, 30 and 32, Sardinia Street, Lincoln's Inn Fields, London, W.C.

**Cheap Advertisements** of the "Wanted" class, including those for situations, of Twenty-four Words or under, are charged 1*s.* an insertion, or three insertions for 2*s.* 6*d.*, but must be paid for in advance. Each additional line of Eight Words is 4*d.*

**Employers requiring assistants** are charged for one insertion of Twenty-four Words or under, 2*s.* 6*d.*, or 6*s.* for three insertions. Each additional line of Eight Words is 1*s.*

**Advertisements** for insertion in the weekly issue of THE HOSPITAL **must be delivered** at the Publishing Offices **not later than 11 a.m. on Wednesdays.**

### TERMS OF SUBSCRIPTION.

Subscribers to THE HOSPITAL residing in London and the Suburbs will receive their Copies by the first post on FRIDAY MORNING. Copies for Country Subscribers are forwarded by the DAY Mail on Fridays. Terms, including postage, payable in advance:

| | Weekly Parts. | Monthly Parts. |
|---|---|---|
| Twelve months | 0 6 6 | 0 7 6 |
| Six " | 0 3 3 | 0 3 9 |
| Three " | 0 1 8 | 0 1 11 |

Post Office Orders should be made payable at the General Post Office, London, E.C., to WHITING AND Co., 30 and 32, Sardinia Street, Lincoln's Inn Fields, W.C. Cheques to be crossed "London and Joint Stock Bank, Limited," Chancery Lane Branch.

**Cases for Binding** the weekly or monthly parts of THE HOSPITAL in yearly vols. may be ordered of the Publishers.

